# Clinical expert consensus document on intravascular ultrasound from the Japanese Association of Cardiovascular Intervention and Therapeutics (2021)

**DOI:** 10.1007/s12928-021-00824-0

**Published:** 2021-11-12

**Authors:** Yuichi Saito, Yoshio Kobayashi, Kenichi Fujii, Shinjo Sonoda, Kenichi Tsujita, Kiyoshi Hibi, Yoshihiro Morino, Hiroyuki Okura, Yuji Ikari, Junko Honye

**Affiliations:** 1 Department of Cardiovascular Medicine, Chiba University Graduate School of Medicine, 1-8-1 Inohana, Chuo-ku, Chiba, Chiba 260-8677 Japan; 2 Division of Cardiology, Department of Medicine II, Kansai Medical University, Hirakata, Japan; 3 Department of Cardiovascular Failure Therapy, Saga University, Saga, Japan; 4 Department of Cardiovascular Medicine, Kumamoto University, Kumamoto, Japan; 5 Division of Cardiology, Yokohama City University Medical Center, Yokohama, Japan; 6 Department of Cardiology, Iwate Medical University Hospital, Morioka, Japan; 7 Department of Cardiology, Gifu University Graduate School of Medicine, Gifu, Japan; 8 Department of Cardiology, Tokai University Hospital, Isehara, Japan; 9 Department of Cardiovascular Medicine, Kikuna Memorial Hospital, Yokohama, Japan

**Keywords:** Intravascular ultrasound, Coronary artery disease, Definition, Measurement

## Abstract

Intravascular ultrasound (IVUS) provides precise anatomic information in coronary arteries including quantitative measurements and morphological assessment. To standardize the IVUS analysis in the current era, this updated expert consensus document summarizes the methods of measurements and assessment of IVUS images and the clinical evidence of IVUS use in percutaneous coronary intervention.

## Introduction

In 2019, the Japanese Association of Cardiovascular Intervention and Therapeutics (CVIT) published an expert consensus document on intravascular ultrasound (IVUS) [1]. The consensus document in 2019 only focused on standards for measurements and assessment of IVUS. Despite the clinical evidence of IVUS use in percutaneous coronary intervention (PCI), this intracoronary imaging modality may be underused worldwide. This updated expert consensus document adds a brief summary of recent clinical evidence of IVUS in the section VI “Clinical evidence”.

## Principles and precautions

IVUS has become increasingly important in both clinical and research applications. PCI under IVUS guidance has been consistently shown to be superior to angiography-guided PCI [2], and a number of clinical studies have employed IVUS in the field of coronary artery disease [3]. Although the American College of Cardiology Clinical Expert Consensus Document on Standards for Acquisition, Measurement, and Reporting of Intravascular Ultrasound Studies was published in 2001, there are no updated standards for measurement and assessment of IVUS images in the current era [4]. To facilitate to communicate findings using a common language and avoid confounded literature by ambiguous terminology, the present expert consensus document provides a contemporary framework for standardization of IVUS analysis.

The principle of IVUS imaging is based on the oscillatory movement (expansion and contraction) of a piezoelectric transducer (crystal) to produce sound waves when electrically excited. After reflection from tissue, part of the ultrasound energy returns to the transducer, which produces an electrical impulse that is converted into the image. There are two major different transducer designs: (1) the mechanically rotating transducer and (2) the electronic phased array system. The first design uses a single piezoelectric rotating transducer, whereas the latter uses multiple stationary placed piezoelectric transducers which are sequentially activated.

Current IVUS systems operate at frequencies between 20 and 60 MHz. The higher the frequency, the higher the resolution, but the lower the penetration [5]. However, recent improvements in transducer design have minimized the negative impact of higher frequencies on penetration. High-definition IVUS system offers superior axial resolution, faster catheter pullback speed, and rapid image acquisition compared to conventional IVUS. Thus, image acquisition at the higher frequency is encouraged to decrease variability and improve reproducibility for IVUS analysis. Automatic motorized pullback is highly recommended to acquire IVUS images especially in clinical studies, which should start at least 10 mm distal to the region of interest (e.g. target lesion and stented segment) and ideally continue until the aorta is visualized [3]. To interrogate aorto-ostial lesions, the guiding catheter should be disengaged from the ostium. The automatic pullback speed ranges from 0.5 to 10 mm/s in current IVUS systems, but its impact on the measurement and assessment of IVUS images is unknown. Although pullback speed has been usually at a rate of 0.5 or 1 mm/s, a recent study demonstrated good agreement with respect to length measurement between pullback speed of 0.5 and 10 mm/s [6]. Although recently developed IVUS systems are reported to provide comparable 2-dimensional quantitative measurements with each other [7], it is important to employ the same equipment (IVUS catheters, consoles and pullback devices) in clinical investigations [8], especially for baseline and follow-up studies [3]. IVUS examination should be performed with intravenous administration of anticoagulation (e.g. heparin). Intracoronary nitrates should also be routinely administered prior to delivering the IVUS catheter to induce maximal vasodilation and to prevent vasospasm.

There are some known artifacts on IVUS imaging. Non-uniform rotational distortion (NURD) is unique to mechanical catheter systems due to mechanical binding of the drive cable that rotates the transducer, which results in a wedge-shaped, smeared appearance [9]. This phenomenon usually occurs in tortuous vessels and acute bends in the artery. Any cross sections with a recognizable NURD that precludes accurate definition of the leading edge of the outer vessel wall border should be eliminated following the same rules as for calcium. A distinct motion artifact can result from non-stable catheter position with cardiac cycles: (1) in-plane rigid motion and (2) forward and backward longitudinal motion along the catheter axis [10]. The vessel occasionally moves before a complete circumferential image can be created. Any cross sections with severe motion artifacts that preclude accurate definition of the leading edge should be avoided. In addition, an IVUS transducer can longitudinally move as much as 5 mm between diastole and systole [11]. This move has been reduced in a recent modern faster pullback device. Ring-down artifacts are usually observed as bright halos of variable thickness surrounding the IVUS catheter. In the phased array systems, ring-down artifact can be partially reduced by digital subtraction of a reference mask. However, if it is incorrectly performed, digital subtraction has the potential to remove real information or introduce false targets. Side lobes are artifact of extraneous beams of ultrasound that are generated from the edges of the individual transducer elements and are not in the direction of the main ultrasonic beam, originated from a strong reflecting surface, such as metal stent struts and calcification. All artifacts should be taken into account for measurement and assessment of IVUS images.

PCI optimization under IVUS guidance can improve clinical outcomes [12, 13], but IVUS can also provide clarity where the angiogram demonstrates ambiguity, such as: (1) intermediate lesions of uncertain stenotic severity; (2) coronary aneurysm and ectasia; (3) aorto-ostial lesions; (4) disease at branching sites; (5) tortuous/overlapping vessels; (6) left main stem lesions; (7) sites with focal spasm; (8) sites with plaque rupture and/or thrombus; (9) dissections; (10) intraluminal filling defects; (11) extramural hematoma (extravasation); (12) coronary perforation; (13) angiographically hazy lesions; and (14) lesions with local flow disturbances.

## Definition of lesion/stenosis and reference

Since atherosclerosis in coronary arteries often appears to be more extensive by IVUS than by angiography [14], appropriate definitions of lesion and reference segment nomenclature require different methodology than commonly employed in angiography. The following are definitions:

- Proximal reference: The site with the largest lumen proximal to a stenosis but within the same segment (usually within 10 mm of the stenosis with no major intervening branches). Proximal reference may or may not be the site with the least plaque.
- Distal reference: The site with the largest lumen distal to a stenosis but within the same segment (usually within 10 mm of the stenosis with no major intervening branches). This may or may not be the site with the least plaque.
- Largest reference: The largest of either the proximal or distal reference sites.
- Average reference lumen size: The average value of lumen size at the proximal and distal reference sites.
- Lesion: A lesion represents accumulation of atherosclerotic plaque compared to a predefined reference
- Stenosis: A stenosis is a lesion that compromises the lumen by at least 50% by cross-sectional area compared to a predefined reference segment lumen.
- Worst stenosis: The stenosis with the smallest lumen size, which may or may not represent the site with the largest atheroma.
- Secondary stenoses: Lesions meeting the definition of a stenosis, but with lumen sizes larger than the worst stenosis.

The reference segment used for identifying lesions and stenoses should be predefined and specified as proximal, distal, largest, or average of references. In the case of multiple lesions within a single coronary segment, distinct lesions or stenoses require at least 5 mm between them. If not, the disease should be considered as a single lesion. Efforts should be made to use the same reference sites before and after intervention especially in serial studies in which the same anatomic images will be measured and compared (e.g. pre- vs. post-intervention or post-intervention vs. follow-up), although the location of the smallest lumen may or may not be different at each time point. The following sequence can be used to identify image slices on serial studies: (1) an image slice is selected from the first study, and the distance from this image slice to the closest identifiable axial landmark (a fiduciary point) (e.g. side branches and calcific deposits) is measured; (2) the second study is screened to identify this fiduciary point, and the previously measured distance is used to identify the corresponding image slices on the second study; (3) vascular and axial landmarks are used to confirm slice identification. Studies should be analyzed side by side and the imaging runs studied frame by frame if necessary. To assess the morphology of the lesion or stenosis (e.g. plaque composition or calcium), the entire lesion or stenosis should be surveyed, not just the worst stenosis image.

## Quantitative measurements

**Fig. 1:**
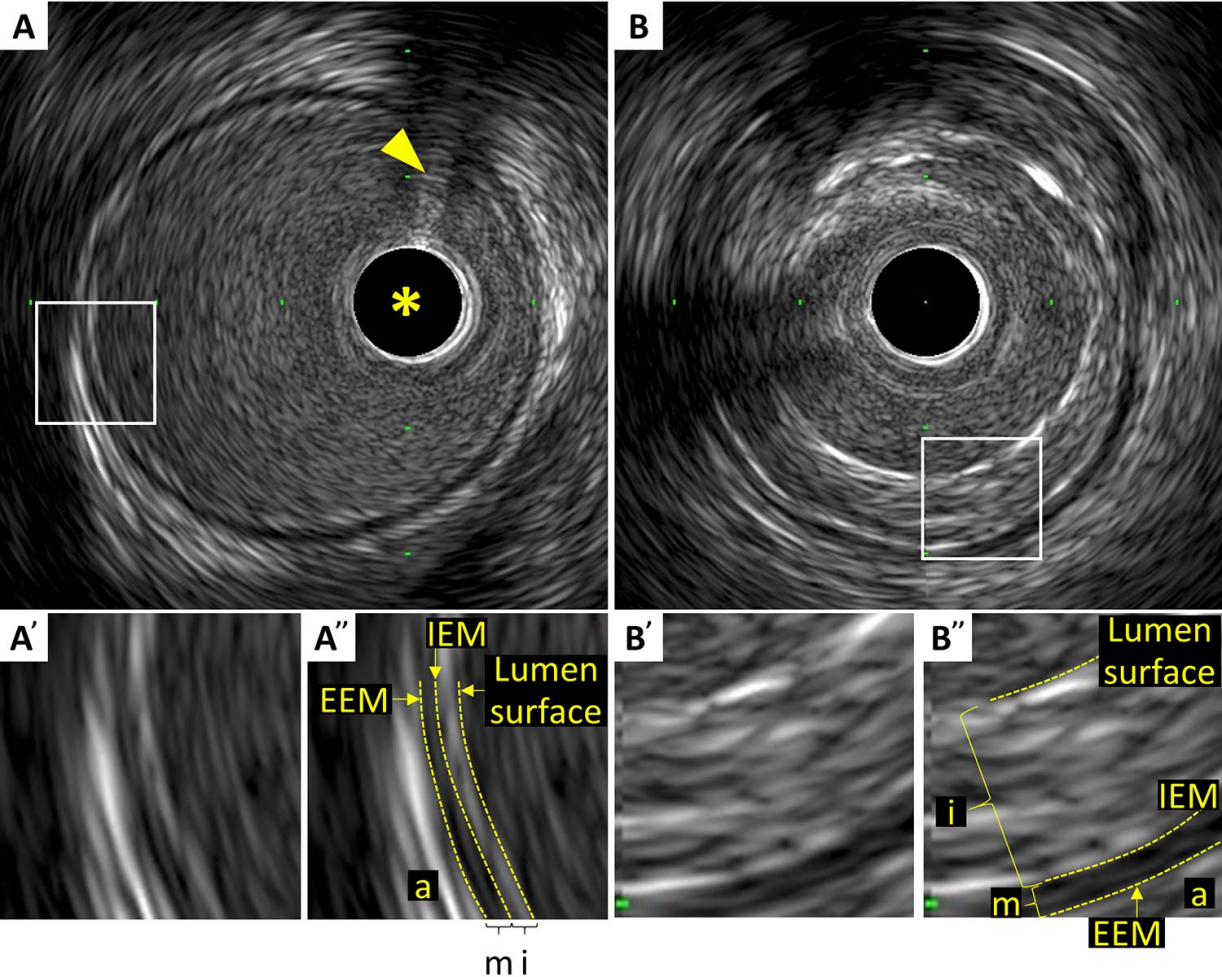
Three layers of coronary arteries on intravascular ultrasound. Coronary arteries consist of three layers (“i”—intima, “m”—media, “a”—adventitia). The innermost layer, tunica intima, comprises three elements: endothelium (corresponding to the lumen surface), atheroma (if the arteries are diseased), and internal elastic membrane (IEM). The second layer is tunica media, which consists of smooth muscle and external elastic membrane (EEM). The third and outer layer is tunica externa, which comprises the adventitia and peri-adventitial tissues. **A** Normal coronary artery. **B** Diseased vessel. Asterisk indicates intravascular ultrasound catheter, and arrowhead indicates guidewire with acoustic shadow

In muscular arteries including coronary arteries, there are three layers (Fig. 1) [15]. The innermost layer, tunica intima, comprises a complex of three elements: endothelium, atheroma (if the arteries are diseased), and internal elastic membrane. This layer is highly echogenic compared to the lumen and media. The trailing edge of the intima (which would be corresponding to the internal elastic membrane) cannot always be distinguished clearly. The second layer is tunica media, which consists of smooth muscle and external elastic membrane (EEM). The third and outer layer is tunica externa, which comprises the adventitia and peri-adventitial tissues. There is no distinct boundary on IVUS images separating the true adventitia from surrounding perivascular tissues. The vasa vasorum, a network of small blood vessels that supply the coronary vessel wall can occasionally be visualized on IVUS image exterior to media [16].

In the cross-sectional analysis, measurements should be avoided if significant artifacts, such as NURD and motion artifact, are present, if the IVUS catheter is obliquely positioned, or if large side branches originate. Area measurements can be added to calculate volumes with the Simpson’s Rule. Analysis routinely subsamples at predefined intervals, typically every 1 mm. All tracing and measurements should be performed at the leading edge of boundaries, never the trailing edge (Fig. 1). Measurements at the trailing edge are inconsistent and frequently result in erroneous results.

### Lumen measurements

**Fig. 2:**
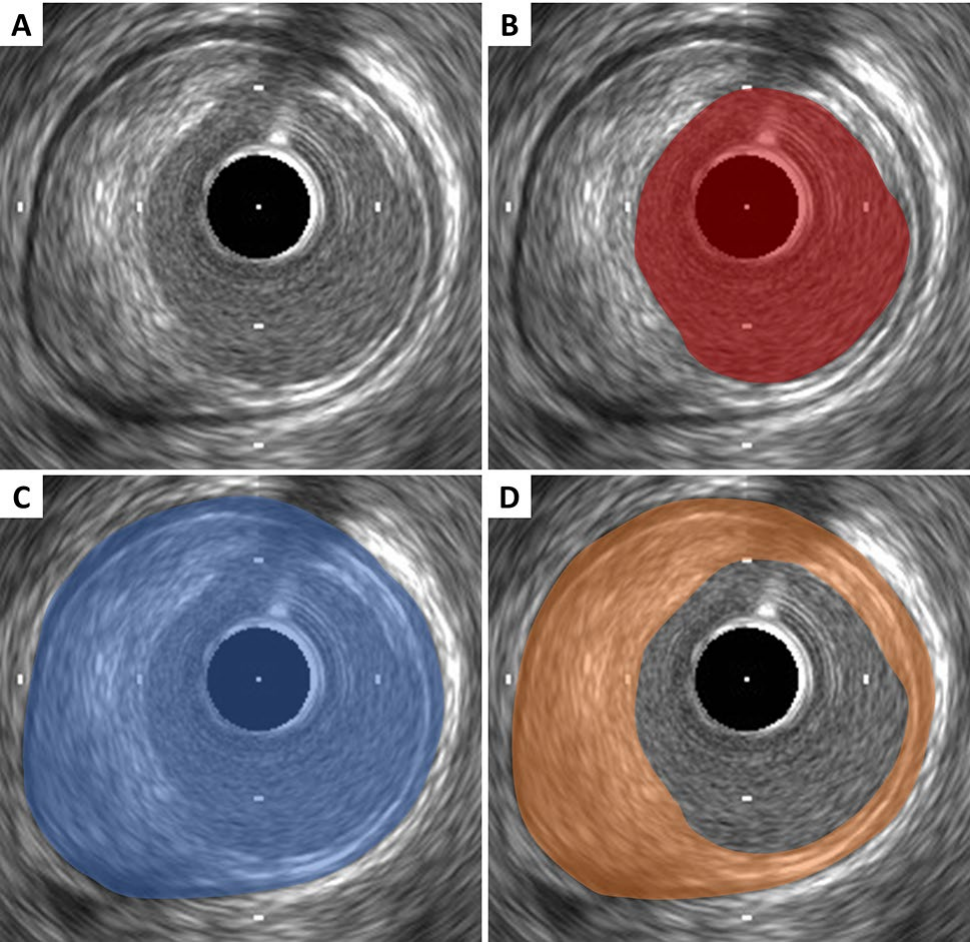
Lumen, external elastic membrane (EEM), and plaque area measurements. **A** Coronary cross-sectional image with atheromatous plaque. **B** Lumen area is measured by tracing the leading edge of the intima. **C** EEM area is measured by tracing the border between the media and the adventitia. **D** Plaque area is calculated by (EEM area) − (lumen area)

Lumen measurements are performed using the interface between the lumen and the leading edge of the intima (Fig. 2). The leading edge of the innermost echogenic layer is usually used as the lumen boundary. Once the lumen border is determined, the following measurements can be derived. In all cases, measurements are performed relative to the center of the lumen rather than relative to the center of the IVUS catheter.

- Lumen area: The area bounded by the luminal border (Fig. 2).
- Minimum lumen diameter: The shortest diameter through the center point of the lumen.
- Maximum lumen diameter: The longest diameter through the center point of the lumen.
- Lumen eccentricity: [(Maximum lumen diameter − minimum stent diameter)/maximum lumen diameter].
- Lumen area stenosis: [(Reference lumen area – minimum lumen area [MLA])/reference lumen area]. The reference segment used for the calculation should be predefined and specified (proximal, distal, largest, or average).

If dissection is present, whether the lumen area is the true lumen or a combination of the true and false lumen should be reported.

### External elastic membrane measurement

A discrete interface at the border between the media and the adventitia is usually present on IVUS images, and almost corresponds to the location of the EEM (Fig. 1). The EEM area is the recommended term for this measurement, but alternative terms such as “vessel area” are often used. Tracing EEM (the leading edge of adventitia) includes lumen, intima with atheroma (in diseased arteries) and media, but adventitia. EEM circumference cannot be reliably identified at sites where large side branches originate or acoustic shadowing by extensive calcification presents. If acoustic shadowing involves a relatively small arc < 90°, EEM measurement can be performed by extrapolation from the closest identifiable EEM borders. If calcification is more extensive than 90° of arc, EEM measurements should be avoided. Disease-free coronary arteries are circular, but atherosclerotic arteries may remodel into a non-circular configuration. If maximum and minimum EEM diameters are reported, measurements should bisect the geometric center of the vessel rather than the center of the IVUS catheter.

- EEM area: The area bounded by the leading edge of adventitia (Fig. 2).
- Minimum EEM diameter: The shortest diameter through the center point of the vessel.
- Maximum EEM diameter: The longest diameter through the center point of the vessel.

### Plaque measurement

Since the leading edge of the media (corresponding to the internal elastic membrane) is not delineated well, IVUS measurements cannot determine true histological atheroma area (the area bounded by the internal elastic membrane). Accordingly, the EEM and lumen areas are used to calculate a surrogate for true plaque area in IVUS studies. Because the media represents only a very small fraction of the plaque area, including the media into the plaque area does not constitute a major limitation of IVUS in practice. The term “plaque plus media area” is correct and recommended, although alternative terms such as “plaque area” are often used.

- Plaque plus media area: The EEM area − the lumen area (Fig. 2).
- Minimum plaque plus media thickness: The shortest distance form intimal leading edge to the EEM along any line passing through the center of the lumen.
- Maximum plaque plus media thickness: The longest distance form intimal leading edge to the EEM along any line passing through the center of the lumen.
- Plaque plus media eccentricity: [(Maximum plaque plus media thickness − minimum plaque plus media thickness)/maximum plaque plus media thickness].
- Plaque burden: Plaque plus media area divided by the EEM area. This measurement is independent on the lumen area stenosis. The plaque burden represents the area within the EEM occupied by plaque regardless of lumen compromise.

### Stent measurements

**Fig. 3:**
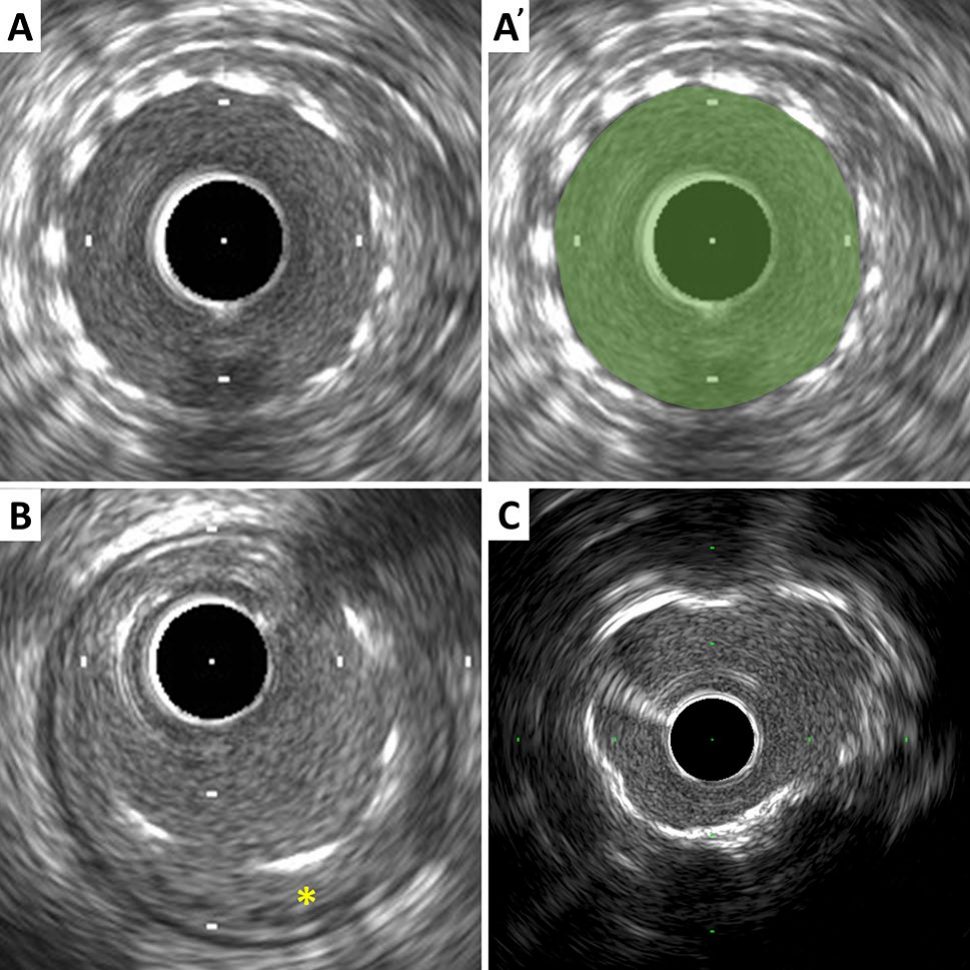
Stent and calcium assessment. **A** Stent area is measured by tracing the leading edge of the stent strut. **B** Incomplete stent apposition (mal-apposition). Blood flow between struts and the underlying wall is observed (asterisk). **C** Calcium appears as bright echoes with acoustic shadowing, which may also produce reverberations or multiple reflections

Metallic stent struts are strong reflectors of ultrasound, thus appear as high echogenic points or arcs along circumference of the vessel (Fig. 3). Strut apposition refers to the proximity of stent struts to the arterial wall [17]. Good apposition is defined as sufficiently close contact to preclude blood flow between any strut and the underlying wall (Fig. 3). If necessary, flushing contrast or saline can enhance to confirm the presence or absence of flow behind the strut. The arc and/or length of incomplete apposition, which is also called as mal-apposition, can be reported. The metallic struts easily create side lobes, which may obscure the true lumen and stent borders and interfere with area measurements and the assessment of apposition, dissection, etc. The stent area is measured by planimetry of the area bounded by the leading edge of stent struts. If strut incomplete apposition is present, the stent area will be smaller than the lumen area. In the case of previously placed stents with superimposed neo-intimal proliferation, the stent area will be larger than the lumen area. In serial studies (post-intervention vs. follow-up), intimal hyperplasia and chronic stent recoil can be assessed.

- Stent area: The area bounded by stent border (leading edge of struts) (Fig. 3).
- Minimum stent diameter: The shortest diameter through the center of mass of the stent.
- Maximum stent diameter: The longest diameter through the center of mass of the stent.
- Stent symmetry: [(Maximum stent diameter − minimum stent diameter)/maximum stent diameter].
- Stent expansion: The minimum stent area compared to the predefined reference area (proximal, distal, largest, or average). Stent under expansion is an area of inadequately expanded stent compared to the adjacent normal reference segment, usually defined by stent expansion < 80% of the reference vessels or a single cut-off value of minimum stent area (e.g. < 5.0 or 5.5 mm^2^) [13, 18, 19].
- Neointima: Previously placed stent area − lumen area.
- Chronic stent recoil: Post-implantation minimum stent area at baseline is compared to minimum stent area at follow-up.

### Calcium measurements

IVUS is a sensitive in vivo method for detecting coronary calcium. Calcific deposits appear as bright echoes that obstruct the penetration of ultrasound, a phenomenon known as acoustic shadowing (Fig. 3). Thus, IVUS can detect only the leading edge and cannot determine the thickness of the calcium. Calcium may also produce reverberations or multiple reflections that result from the oscillation of ultrasound between transducer and calcium and cause concentric arcs in the image at reproducible distances. Calcium deposits are described semi-quantitatively according to their location and distribution.

- Superficial calcium: The leading edge of the acoustic shadow appears within the most shallow 50% of the plaque plus media thickness.
- Deep calcium: The leading edge of the acoustic shadow appears within the deepest 50% of the plaque plus media thickness.

Arc of calcium can be measured using an electronic protractor centered on the lumen. The length of the calcific deposit can also be measured using motorized transducer pullback. Spotty calcium, a lesion that contained only small calcium deposits within an arc of less than 90°, can be assessed, which is more likely to be found in culprit lesions in patients with myocardial infarction (MI) than those with stable coronary artery disease [20].

### Reference segment measurements

Once the reference segments are selected, quantitative and qualitative assessment similar to the stenosis should be performed in both proximal and distal reference, including lumen, EEM, and plaque measurements.

### Remodeling

Vascular remodeling refers to the increase or decrease in EEM area which occurs during the development of atherosclerosis [21]. IVUS imaging allows in vivo assessment of vascular remodeling.

Remodeling is traditionally assessed by comparing lesion site EEM area with reference segment EEM area. Reference EEM area is usually calculated as the average value of EEM size at the proximal and distal reference sites [22], but proximal reference area can be also used as reference EEM area in cases with acute coronary syndrome (ACS) [23]. An index that describes the magnitude and direction of remodeling is expressed as: lesion EEM area/reference EEM area. If the lesion EEM area is greater than the reference EEM area, which represents positive remodeling, the index will be > 1.0. However, such a static definition is not recommended. Instead, remodeling should be assessed in serial studies as: EEM area at follow-up/EEM area at baseline [24]. The index > 1.0 represents positive remodeling, while positive or negative remodeling is often defined as > 5% or 10% increase or decrease of the index [25, 26]. Vessel segments with positive remodeling should be sub-divided as expansive (over compensatory) or incomplete. Furthermore, for IVUS studies that assess progression and regression of coronary atherosclerosis based on serial imaging, following indices can be endpoints.

- Positive remodeling: EEM area at follow-up/EEM area at baseline > 1.0.
- Negative remodeling: EEM area at follow-up/EEM area at baseline < 1.0.
- Expansive positive remodeling: (EEM area at follow-up − EEM area at baseline)/(plaque area at follow-up − plaque area at baseline) > 1.0.
- Incomplete positive remodeling: (EEM area at follow-up − EEM area at baseline)/(plaque area at follow-up − plaque area at baseline) < 1.0.
- Total atheroma volume (TAV): Σ (EEM area − lumen area).
- Normalized TAV: (TAV × mean or median no. of analyzed frames in the population)/no. of analyzed frames per patients.
- Percentage of change in TAV: [(TAV at follow-up − TAV at baseline)/TAV at baseline] × 100.
- Percent atheroma volume (PAV): [Σ (EEM area − lumen area)/Σ EEM area] × 100.
- The absolute change in PAV: PAV at follow-up − PAV at baseline.

### Length

Length measurements by IVUS can be performed using motorized transducer pullback system (number of seconds × pullback speed). Alternatively, longitudinal imaging can be used. In case with manual pullback without motorized transducer pullback system, length cannot be measured. This approach can be used to determine the length of a lesion, stenosis, calcium, or any other longitudinal feature.

## Qualitative measurements

### Plaque morphology

**Fig. 4:**
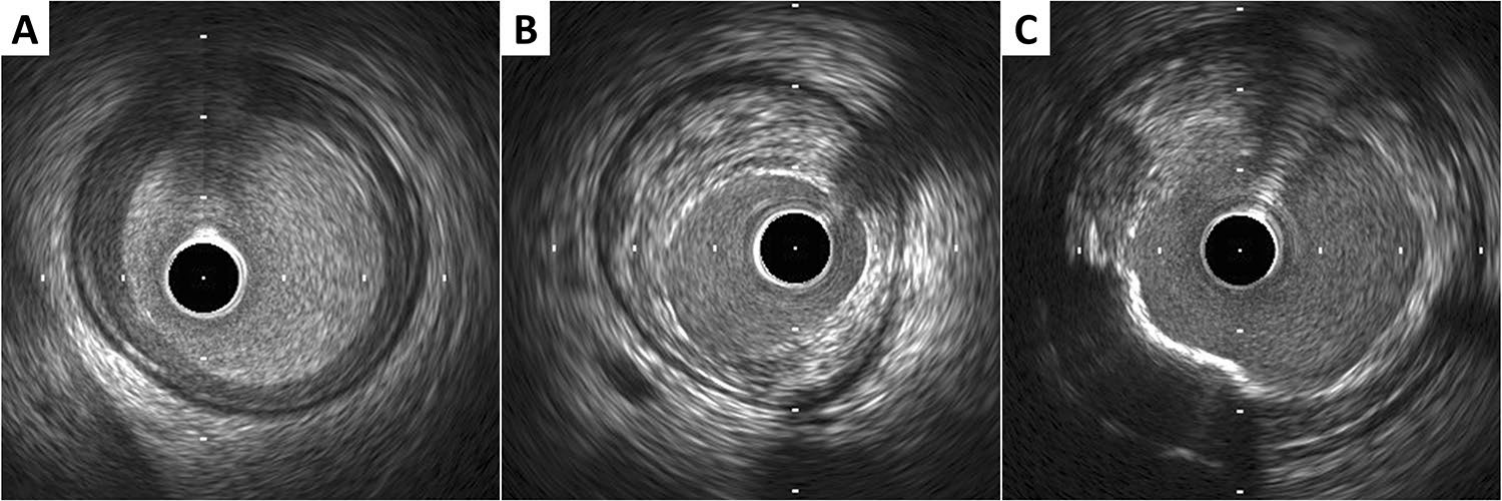
Representative images of soft (lipid), fibrous, and mixed plaque. **A** Soft (lipid) plaque has low echogenicity in contrast to the reference adventitia. **B** Fibrous plaque has an intermediate echogenicity between soft (hypoechoic) plaque and highly echogenic calcified plaques. **C** Mixed plaque contains more than one subtype

Ultrasound images are fundamentally different from histology, thus gray-scale IVUS cannot specify and quantify histologic contents. However, tissue characterization technologies, such as virtual histology IVUS (VH-IVUS, Volcano Therapeutics, Rancho Cordova, USA), integrated backscatter IVUS (IB-IVUS, Terumo, Tokyo, Japan), and iMAP-IVUS (Boston Scientific, Santa Clara, USA), have partially overcome this limitation. In addition, the recent combination of near-infrared spectroscopy (NIRS) with IVUS in a single imaging catheter allows simultaneous assessment of plaque composition, specifically to quantify lipid plaque content. Qualitative plaque characteristics by gray-scale IVUS are defined as follows:

- Soft (lipid) plaque: The term “soft” refers to the acoustic signal which arises from low echogenicity in contrast to the reference adventitia, rather than plaque’s structural characteristics (Fig. 4). In general, this is the result of high lipid content in a mostly cellular lesion. However, a zone of reduced echogenicity may also be attributable to a necrotic zone within the plaque, an intramural hemorrhage, or a thrombus. Most soft plaques contain minimal collagen and elastin.
- Fibrous plaque: This has an intermediate echogenicity between soft (hypoechoic) plaque and highly echogenic calcified plaques (Fig. 4). Fibrous plaque mainly represents atherosclerotic lesions. In general, the greater the fibrous tissue content, the higher the echogenicity of the tissue.
- Calcified plaque: See section on calcium measurement.
- Mixed plaque: Plaques containing more than one subtype (Fig. 4). There are a number of terminology for these plaques, such as “fibrocalcific” and “fibrofatty”.
- Intimal hyperplasia: Neo-intimal growth after bare metal stent (BMS) implantation has an early peak, whereas late neo-intimal growth develops within drug-eluting stent (DES). The morphology of intimal hyperplasia can be also assessed as plaque characteristics (soft/lipid, fibrous, calcified, and mixed). The neointima with accumulation of lipid plaques with or without calcification inside previously implanted stents is called “neoatherosclerosis” [27, 28].

**Fig. 5:**
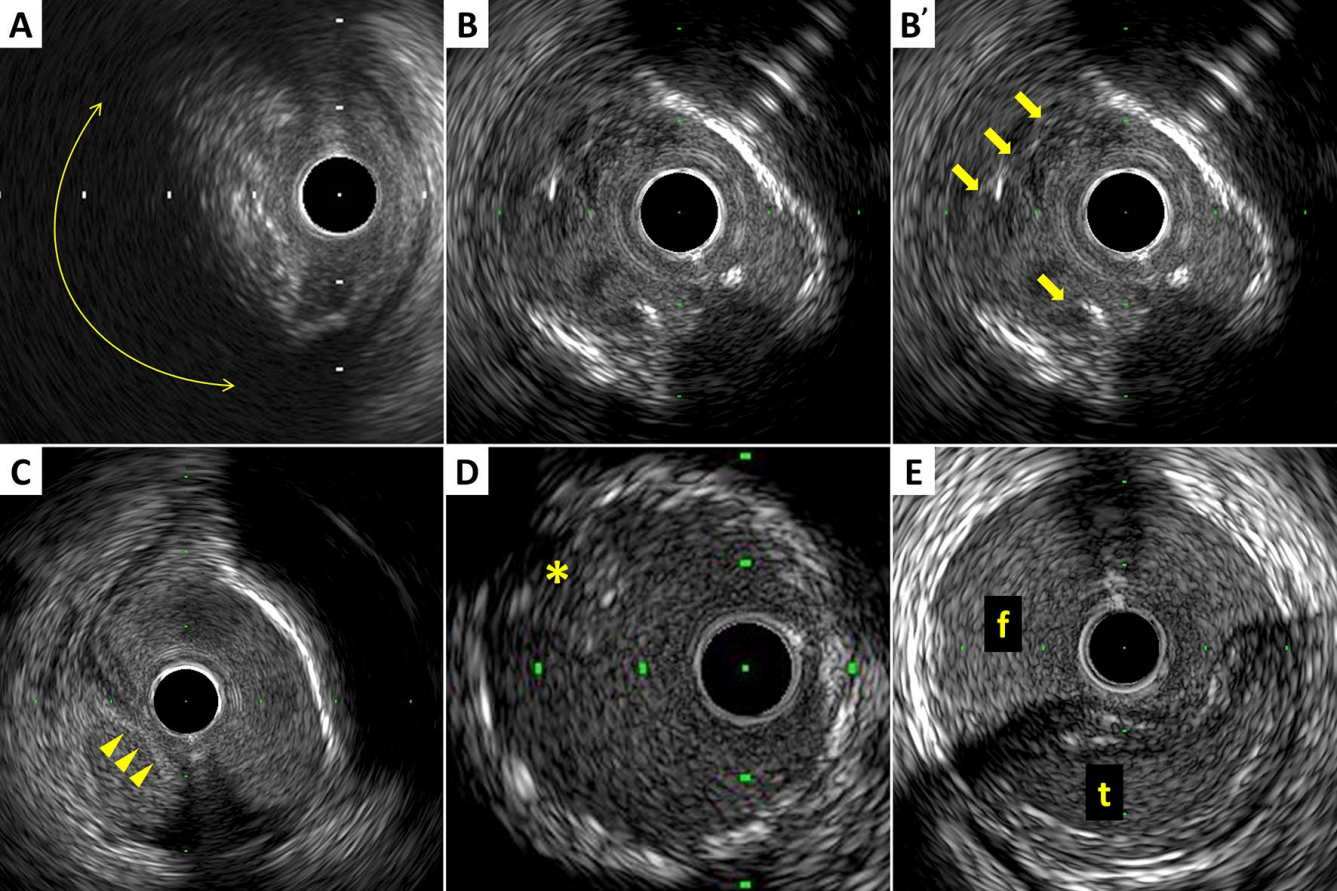
Examples of qualitative measurements. **A** Attenuated plaque is accompanied by backward signal attenuation without dense calcium (circular arc). **B** Thrombus is usually observed as an intraluminal mass, often with a layered, lobulated, or pedunculated appearance (arrows). **C** Dissection with mobile flap (arrowheads). **D** Tissue protrusion is detected as tissue extrusion from stent or scaffold (asterisk). **E** True lumen (“t” in image) is surrounded by all three layers of the vessel wall, whereas false lumen (“f” in image) is not

The plaque accompanied by backward signal attenuation without dense calcium is called “attenuated plaque” (Fig. 5). Attenuated plaque is associated with a large amount of necrotic core (i.e. vulnerable plaque) [29], leading to no-flow phenomenon during PCI and subsequent coronary events [30, 31].

### Thrombus

A thrombus is usually recognized as an intraluminal mass, often with a layered, lobulated, or pedunculated appearance on IVUS (Fig. 5) [32]. Thrombi may appear relatively hypoechoic or have a more variable gray scale with speckling or scintillation. Blood flow in “micro-channels” may also be apparent within some thrombi. Injection of contrast or saline may disperse the stagnant flow, clear the lumen, and allow differentiation of stasis from thrombosis. However, the diagnosis of thrombus by IVUS may be compromised and should always be considered presumptive.

### Dissection

Dissection is observed as a complication of PCI or as spontaneous coronary artery dissection (Fig. 5). IVUS can detect dissections during PCI, which are usually within the ballooned segment or on the edge of stents. The dissection is classified into 5 categories as following:

- Intimal: Limited to the intima or atheroma, and not extending to the media.
- Medial: Extending into the media.
- Adventitial: Extending through the EEM (extramural hematoma, extravasation).
- Intramural hematoma: An accumulation of blood within the medial space, displacing the internal elastic membrane inward and EEM outward. Entry and/or exit points may or may not be observed.
- Intra-stent: Separation of neo-intimal hyperplasia from stent struts, usually seen only after treatment of in-stent restenosis.

The severity of a dissection can be quantified according to: (1) depth (into plaque; useful only in describing intimal dissections that do not reach the media); (2) circumferential extent (in degrees of arc) using an electronic protractor centered on the lumen; (3) length (by motorized transducer pullback; (4) size of residual lumen area; and (5) luminal dissection area. Additional descriptions of dissection may include the presence of a false lumen, the identification of mobile flap(s), the presence of calcium at the dissection border, and dissections in close proximity to stent edges. Some dissections may not be apparent by IVUS, because of the scaffolding by the imaging catheter or because the dissection is located behind calcium. If feasible, injection of contrast or saline/glucose solution can enhance the diagnostic capability to detect dissection on IVUS [33].

### Lesion morphology identification in acute coronary syndrome

No definitive IVUS features define a plaque as vulnerable to induce future event, although plaque burden ≥ 70%, MLA < 4 mm^2^ and thin-cap fibroatheroma derived by VH-IVUS were shown to be independent predictors [34]. IB-IVUS is also reported to be useful in predicting ACS [30]. Pathological studies revealed that unstable coronary lesions are usually lipid-rich with a thin fibrous cap [35]. Morphologically, large hypoechoic plaques (i.e. soft/lipid plaque) with no well-formed fibrous cap is considered as vulnerable atherosclerotic lesions. Tissue characterization technologies may help to detect unstable and vulnerable plaques.

Three major underlying mechanisms for ACS include plaque rupture, plaque erosion, and calcified nodule [36, 37], although plaque rupture is the most common cause. Ruptured plaques have a highly variable appearance by IVUS. In patients with ACS, IVUS imaging may show an ulceration, often with remnants of the ruptured fibrous cap evident at the edges of the ulcer. A variety of other appearances are common such as fissuring of the plaque surface [38]. In addition, the presence of thrombi may obscure IVUS detection of plaque fissuring or ulceration. The following definitions are used:

- Plaque ulceration: A recess in the plaque beginning at the luminal-intimal border, typically without enlargement of the EEM compared with the reference segment.
- Plaque rupture: A plaque ulceration with a tear detected in a fibrous cap. Injection of contrast or saline may be used to prove and define the communication point.

The low resolution of IVUS precludes the evaluation of plaque erosions, whereas calcified nodule can be identified as distinct calcification with an irregular, protruding and convex luminal surface [37].

### Tissue protrusion

Tissue protrusion, often called as tissue prolapse, is frequently detected by IVUS following stent implantation, especially in unstable lesions (Fig. 5) [39]. This structure is defined as tissue extrusion from inside the stent area, and may include either lesion protrusion or, in context of ACS, protrusion of athero-thrombotic material [13]. Area of tissue protrusion can be measured by surrounding the border of the tissue extrusion. Although the clinical impact of tissue protrusion on IVUS remains unclear [39, 40], a previous report indicated IVUS-detected tissue protrusion as a predictor of subsequent cardiovascular events in patients with ST-segment elevation MI [41].

### Aneurysm and true vs. false lumen

- True aneurysm: A lesion that includes all layers of the vessel wall (i.e. intima, media, and adventitia) with an EEM and lumen area > 50% larger than the proximal reference segment.
- Pseudoaneurysm: Disruption of the EEM, usually observed after intervention.
- True versus false lumen: A true lumen is surrounded by all layers of the vessel wall. Side branched communicate with the true, but not with the false lumen (Fig. 5). A false lumen in a channel, usually parallel to the true lumen, which does not communicate with the true lumen over a position of its length.

### Bioresorbable scaffold

Fully bioresorbable scaffold (BRS) has been designed to provide transient mechanical support against acute recoil and anti-restenotic benefits in the early phase, and then disappear over time to leave behind only the native coronary vessel. Although the Absorb Bioresorbable Vascular Scaffold (Absorb BVS; Abbott Vascular, Santa Clara, USA), the most studied BRS, was withdrawn from the market because of low demand with the higher rate of device-related events than contemporary drug-eluting stent (DES), BRSs have been developing. Most BRSs are made from not metal but poly-l-lactide, thus the appearance of BRS is different from that of metallic stent [42]. The use of 60 MHz high-definition IVUS is recommended to visualize double layers of scaffold struts, while conventional IVUS (i.e. 40 MHz) may not clearly identify struts [6]. All measurements including scaffold area and incomplete apposition are calculated in a similar fashion to stented lesion.

### Vein graft disease

Wall morphology and plaque characteristics of vein grafts are different from those of native coronary arteries. The bypass graft wall has no side branches and is free from the surrounding tissue. In situ veins do not have an EEM. However, vein grafts typically undergo “arterialization” with morphologic changes that include intimal fibrous thickening, medial hypertrophy, and lipid deposition. The EEM area is measured by tracing the outer border of the sonolucent zone [43]. All other measurements including plaque plus media area and plaque burden, are calculated in a similar fashion to native coronary disease.

### Assessment of transplant vasculopathy

Coronary disease represents the major cause of death following transplantation and is often clinically silent because the heart is denervated. IVUS has emerged as the gold standard for early detection of cardiac allograft vasculopathy (CAV). The European guidelines for the management of heart transplant patients recommend utilizing IVUS in conjunction with coronary angiography at baseline and follow-up to detect rapidly progressive CAV [44]. The severity of CAV is classified according to the intimal thickness and its degrees of arc [45]. The vessel wall in post transplantation patients may have a single-layer appearance because the intima cannot be resolved as a discrete layer. In such cases, a thin, inner hypoechoic band corresponding to the intima and media is usually present and it is this boundary that should be measured. Although lumen boundaries defined in this manner may include the intima, the thickness of this layer is negligible.

## Clinical evidence

In the era of BMS, randomized control trials (RCTs) showed better clinical outcomes in patients treated under IVUS guidance compared with angiographic guidance alone in PCI [46, 47]. In the DES era, 10 RCTs have compared IVUS-guided with angiography-guided PCI to date, among which the largest ULTIMATE trial revealed that IVUS-guided DES implantation as compared with those under angiography guidance was associated with significantly lower rates of target vessel failure and stent thrombosis in an “all-comers” setting [48]. The favorable effect of IVUS guidance was maintained during 3-year follow-up after PCI in this trial [49]. The ULTIMATE trial also reinforced the fact that optimal PCI results on IVUS (e.g. MLA in the stented segment > 5.0 mm^2^ or > 90% of the MLA at the distal reference segments, plaque burden < 50% at stent edges, and no edge dissection) were significantly associated with better outcomes [48, 49]. A meta-analysis which included the 10 RCTs showed that the routine use of IVUS during coronary DES implantation in addition to angiography improves clinical outcomes including cardiovascular mortality [50]. Another recent meta-analysis which included RCTs and observational studies in the era of BMS and DES also demonstrated that the risks of cardiovascular death (risk ratio [RR] 0.63, 95% confidence interval [CI] 0.54–0.73), MI (RR 0.71, 95% CI 0.58–0.86), stent thrombosis (RR 0.57, 95% CI 0.41–0.79), and target lesion revascularization (RR 0.81, 95% CI 0.74–0.94) were all significantly lower in the IVUS guidance group than in the angiography guidance group [51]. In addition, the contemporary Medicare data in the US confirmed that the use of IVUS was associated with lower long-term morality [52]. On the other hand, the real-world US data also showed that the use of IVUS guidance in PCI remains low (6.9% in 2017) [52]. It is well known that in Japan, intracoronary imaging including IVUS and optical coherence tomography is routinely used during PCI procedures in most cases, against the situations in the US and European countries [53, 54]. NIRS-IVUS has recently emerged as a unique intracoronary imaging system to detect high-risk lipid-rich plaques and to predict coronary events [55, 56]. Given the robust evidence to support routine use of IVUS for improving clinical outcomes, it is conceivable that IVUS is underused globally. Of note, previous health economic analyses indicated that although the IVUS use in PCI is associated with increased upfront costs compared with angiography alone, it is likely to be the cost-effective strategy because of a reduced risk of clinical events [57, 58]. Because not only cost issues but also the lack of skills and knowledges can be barriers of intracoronary imaging use [53, 59], standardized evaluation systems, as shown in the current document, and training programs are warranted.
