# Correction to: Clinical expert consensus document on intravascular ultrasound from the Japanese Association of Cardiovascular Intervention and Therapeutics (2021)

**DOI:** 10.1007/s12928-021-00834-y

**Published:** 2021-12-18

**Authors:** Yuichi Saito, Yoshio Kobayashi, Kenichi Fujii, Shinjo Sonoda, Kenichi Tsujita, Kiyoshi Hibi, Yoshihiro Morino, Hiroyuki Okura, Yuji Ikari, Junko Honye

**Affiliations:** 1 Department of Cardiovascular Medicine, Chiba University Graduate School of Medicine, 1-8-1 Inohana, Chuo-ku, Chiba, Chiba 260-8677 Japan; 2 Division of Cardiology, Department of Medicine II, Kansai Medical University, Hirakata, Japan; 3 Department of Cardiovascular Failure Therapy, Saga University, Saga, Japan; 4 Department of Cardiovascular Medicine, Kumamoto University, Kumamoto, Japan; 5 Division of Cardiology, Yokohama City University Medical Center, Yokohama, Japan; 6 Department of Cardiology, Iwate Medical University Hospital, Morioka, Japan; 7 Department of Cardiology, Gifu University Graduate School of Medicine, Gifu, Japan; 8 Department of Cardiology, Tokai University Hospital, Isehara, Japan; 9 Department of Cardiovascular Medicine, Kikuna Memorial Hospital, Yokohama, Japan

## Correction to: Cardiovascular Intervention and Therapeutics 10.1007/s12928-021-00824-0

The article “Clinical expert consensus document on intravascular ultrasound from the Japanese Association of Cardiovascular Intervention and Therapeutics (2021)”, written by Yuichi Saito, Yoshio Kobayashi, Kenichi Fujii, Shinjo Sonoda, Kenichi Tsujita, Kiyoshi Hibi, Yoshihiro Morino, Hiroyuki Okura, Yuji Ikari and Junko Honye, was originally published electronically on the publisher’s internet portal on 12 November 2021 without open access. With the author(s)’ decision to opt for Open Choice the copyright of the article changed on 9 December 2021 to © The Author(s) 2021 and the article is forthwith distributed under a Creative Commons Attribution 4.0 International License, which permits use, sharing, adaptation, distribution and reproduction in any medium or format, as long as you give appropriate credit to the original author(s) and the source, provide a link to the Creative Commons licence, and indicate if changes were made. The images or other third party material in this article are included in the article’s Creative Commons licence, unless indicated otherwise in a credit line to the material. If material is not included in the article’s Creative Commons licence and your intended use is not permitted by statutory regulation or exceeds the permitted use, you will need to obtain permission directly from the copyright holder. To view a copy of this licence, visit http://creativecommons.org/licenses/by/4.0.

